# Restoration of type I interferon signaling in intrahepatically primed CD8^+^ T cells promotes functional differentiation

**DOI:** 10.1172/jci.insight.145761

**Published:** 2021-02-08

**Authors:** Keigo Kawashima, Masanori Isogawa, Masaya Onishi, Ian Baudi, Satoru Saito, Atsushi Nakajima, Takashi Fujita, Yasuhito Tanaka

**Affiliations:** 1 Department of Virology and Liver Unit, Nagoya City University Graduate School of Medical Sciences, Nagoya, Japan.; 2 Department of Gastroenterology and Hepatology, Yokohama City University School of Medicine, Yokohama, Japan.; 3 Department of Immunology, National Institute of Infectious Diseases, Tokyo, Japan.; 4 Department of Gastroenterology/Internal Medicine, Gifu University Graduate School of Medicine, Gifu, Japan.; 5 Laboratory of Molecular Genetics, Institute for Frontier Life and Medical Science, and; 6 Laboratory of Molecular and Cellular Immunology, Graduate School of Biostudies, Kyoto University, Kyoto, Japan.; 7 Department of Gastroenterology and Hepatology, Graduate School of Medical Sciences, Kumamoto University, Kumamoto, Japan.

**Keywords:** Immunology, Infectious disease, Cellular immune response, Hepatitis, T cells

## Abstract

Hepatitis B virus–specific (HBV-specific) CD8^+^ T cells fail to acquire effector functions after priming in the liver, but the molecular basis for the dysfunction is poorly understood. By comparing the gene expression profile of intrahepatically primed, dysfunctional HBV-specific CD8^+^ T cells with that of systemically primed, functional effector counterparts, we found that the expression of interferon-stimulated genes (ISGs) is selectively suppressed in the dysfunctional CD8^+^ T cells. The ISG suppression was associated with impaired phosphorylation of STAT1 in response to IFN-α treatment. Importantly, a strong induction of type I interferons (IFN-Is) in the liver facilitated the functional differentiation of intrahepatically primed HBV-specific CD8^+^ T cells in association with the restoration of ISGs’ expression in the T cells. These results suggest that intrahepatic priming suppresses IFN-I signaling in CD8^+^ T cells, which may contribute to the dysfunction. The data also suggest a therapeutic value of the robust induction of intrahepatic IFN-Is for the treatment of chronic HBV infection.

## Abstract 2

Functional defects of hepatitis B virus–specific CD8^+^ T cells correlate with impairment of type I interferon signaling after intrahepatic priming.

## Graphical abstract

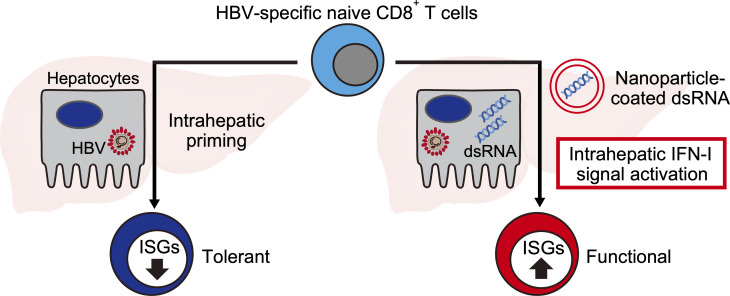

## Introduction

CD8^+^ T cell responses play an essential role in terminating viral infections. During persistent infections, however, virus-specific CD8^+^ T cells progressively lose functionality due to continuous antigen exposure (1). These dysfunctional CD8^+^ T cells, often described as “exhausted,” are characterized by the upregulation of various checkpoint markers, such as programmed cell death 1 (PD-1), T cell immunoglobulin and mucin-domain containing-3 (Tim-3), and lymphocyte-activation gene 3 (Lag-3) (2). The nature of T cell exhaustion has been widely studied during persistent lymphocytic choriomeningitis virus (LCMV) infections (3, 4), a prototype of systemic RNA viral infection. Studies that examined transcriptional signatures of T cells revealed significant differences between effector, memory, and exhausted T cells in the expression of various molecules, such as inhibitory and costimulatory receptors, transcription factors, and signaling molecules (1, 3). Although these pioneering studies have established a notion that exhausted T cells acquire a unique differentiation state, underlying mechanisms for T cell dysfunction likely differ between systemic and local infections because the nature of antigen-presenting cells is very different. In addition, DNA and RNA viruses probably create a very different immunological milieu for T cells.

Hepatitis B virus (HBV) is a partially double-stranded DNA virus that causes acute and chronic infections, as well as hepatocellular carcinoma. The clearance of HBV is dependent on functional virus-specific CD8^+^ T cell responses, which are quantitatively and qualitatively exhausted during persistent HBV infections (5, 6). Early immunological events that lead to HBV-specific T cell exhaustion remain incompletely understood. By adoptively transferring HBV-specific naive T cells into HBV-transgenic (HBV-Tg) mice that replicate HBV in the liver, we have shown previously that HBV-specific CD8^+^ T cells are primed in the liver by HBV-replicating hepatocytes and proliferate in situ (7). However, the intrahepatically primed T cells do not produce interferon-γ (IFN-γ) or exert cytotoxicity. Genetic ablation of PD-1 in HBV-specific CD8^+^ T cells partially induces the effector functions of intrahepatically primed T cells, but the effect is very modest (7). The result suggests that other mechanisms are operative in the functional defects of intrahepatically primed T cells.

In this study, we compared the gene expression profiles of naive, effector, and dysfunctional HBV-specific CD8^+^ T cells to identify molecular signatures unique for intrahepatically primed, dysfunctional CD8^+^ T cells. The results indicate that type I interferon (IFN-I) signaling is strongly suppressed in the dysfunctional CD8^+^ T cells. Surprisingly, T cell receptor (TCR) signaling appears to inhibit the expression of IFN-stimulated genes (ISGs) after IFN-α treatment. Finally, we show that strong induction of IFN-Is in the liver restores the expression of ISGs in the intrahepatically primed T cells and stimulates their functional differentiation. These results illustrate the potentially previously unreported negative impact of intrahepatic antigen recognition on IFN-I signaling in HBV-specific CD8^+^ T cells. The results also indicate that restoration of IFN-I signaling in dysfunctional CD8^+^ T cells may represent a new therapeutic target for terminating persistent HBV infection.

## Results

### Genetic signature of intrahepatically primed dysfunctional HBV-specific CD8^+^ T cells.

**Figure 1:**
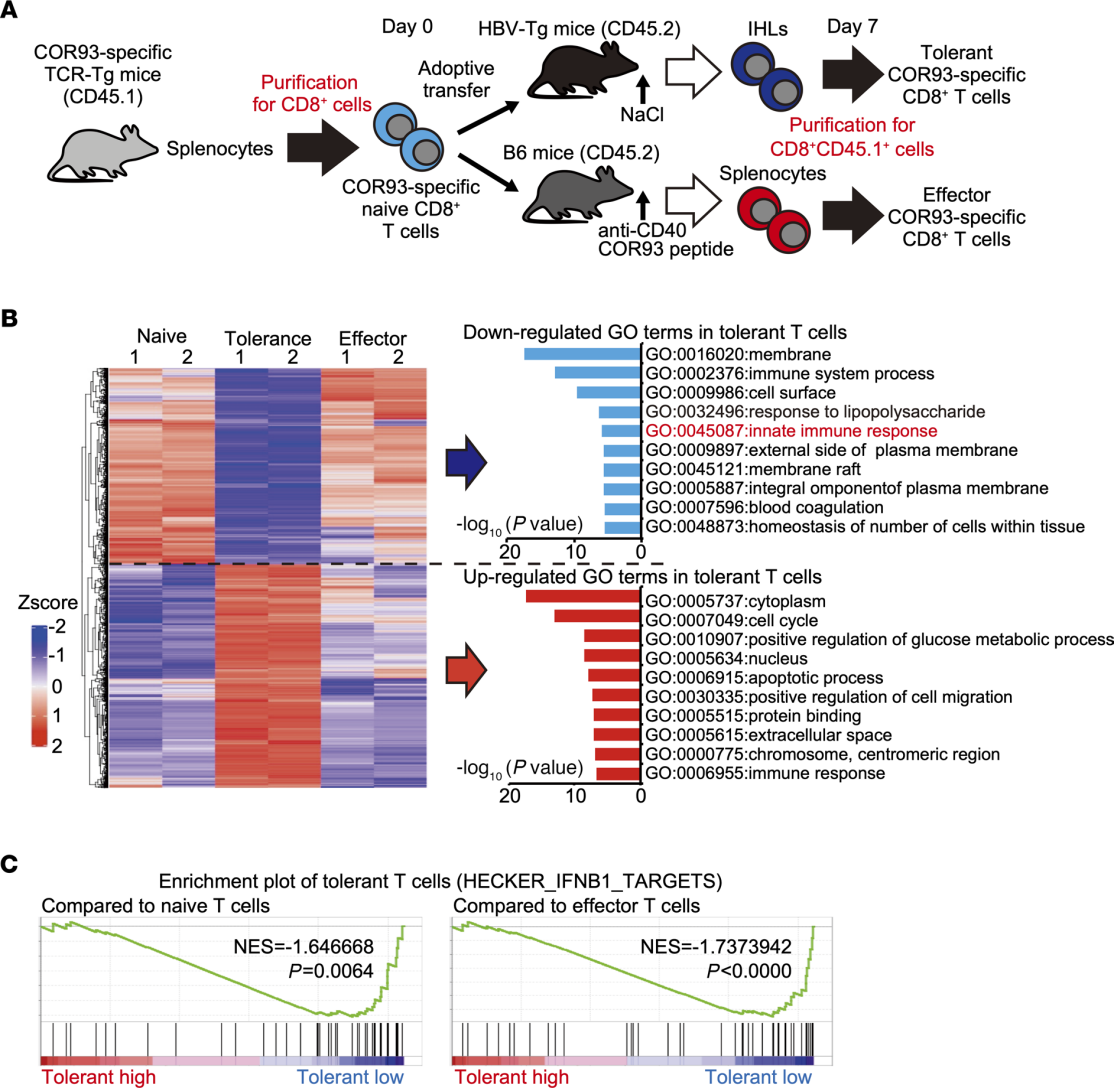
Gene expression profile of intrahepatically primed dysfunctional CD8^+^ T cells. (**A**) Schematic representation of the experiment. (**B**) Left panel: a heatmap of the gene expressions uniquely downregulated or upregulated in tolerant T cells compared with naive and effector T cells (*n* = 2 for each group). Gene ontology (GO) analysis of the down- and upregulated gene sets was performed, and the top 10 GO terms of each set are shown in the right panel. (**C**) Enrichment plots of IFN-I–related gene set (HECKER_IFNB1_TARGET) in tolerant T cells compared with naive (left) or effector (right) T cells.

We have shown previously that intrahepatic antigen recognition by HBV-specific naive T cells results in the expansion of dysfunctional (i.e., tolerant) T cells, while stimulation of the same T cells by dendritic cells induces functional effector T cells (7, 8). To investigate molecular pathways correlated with HBV-specific T cell dysfunction after priming in the liver, we compared the gene expression profiles between tolerant and effector HBV-specific CD8^+^ T cells. Specifically, we adoptively transferred HBV core antigen–derived (HBcAg-derived) peptide–specific (COR93-specific) CD8^+^ T cells into HBV-Tg mice that efficiently replicated HBV in the liver, generating tolerant COR93-specific T cells. To obtain effector COR93-specific T cells, we adoptively transferred the same T cells into C57BL/6 (B6) mice treated with an agonistic anti-CD40 antibody and COR93 peptide (Supplemental Figure 1A; supplemental material available online with this article; https://doi.org/10.1172/jci.insight.145761DS1). Seven days later, adoptively transferred COR93-specific CD8^+^ T cells were purified from intrahepatic lymphocytes (IHLs) of HBV-Tg mice and splenocytes of immunized B6 mice. Donor naive COR93-specific CD8^+^ T cells were also purified for comparison (Supplemental Figure 1B). Gene expression profiles of these T cells were interrogated by microarray analysis (Figure 1A). The data revealed that 2531 genes were uniquely upregulated or downregulated in tolerant T cells compared with naive and effector cells (Supplemental Figure 2A). As shown in Figure 1B, the gene expression profile of tolerant T cells was quite different from that of naive and effector cells. By performing a gene ontology (GO) analysis, we found that genes associated with innate immune response were clearly downregulated in tolerant T cells (Figure 1B). The gene set enrichment analysis (GSEA) also showed that the genes targeted by IFN-β were downregulated in tolerant T cells (Figure 1C and Supplemental Figure 2B).

### Expression of genes related to IFN-I signaling is suppressed in tolerant T cells.

**Figure 2:**
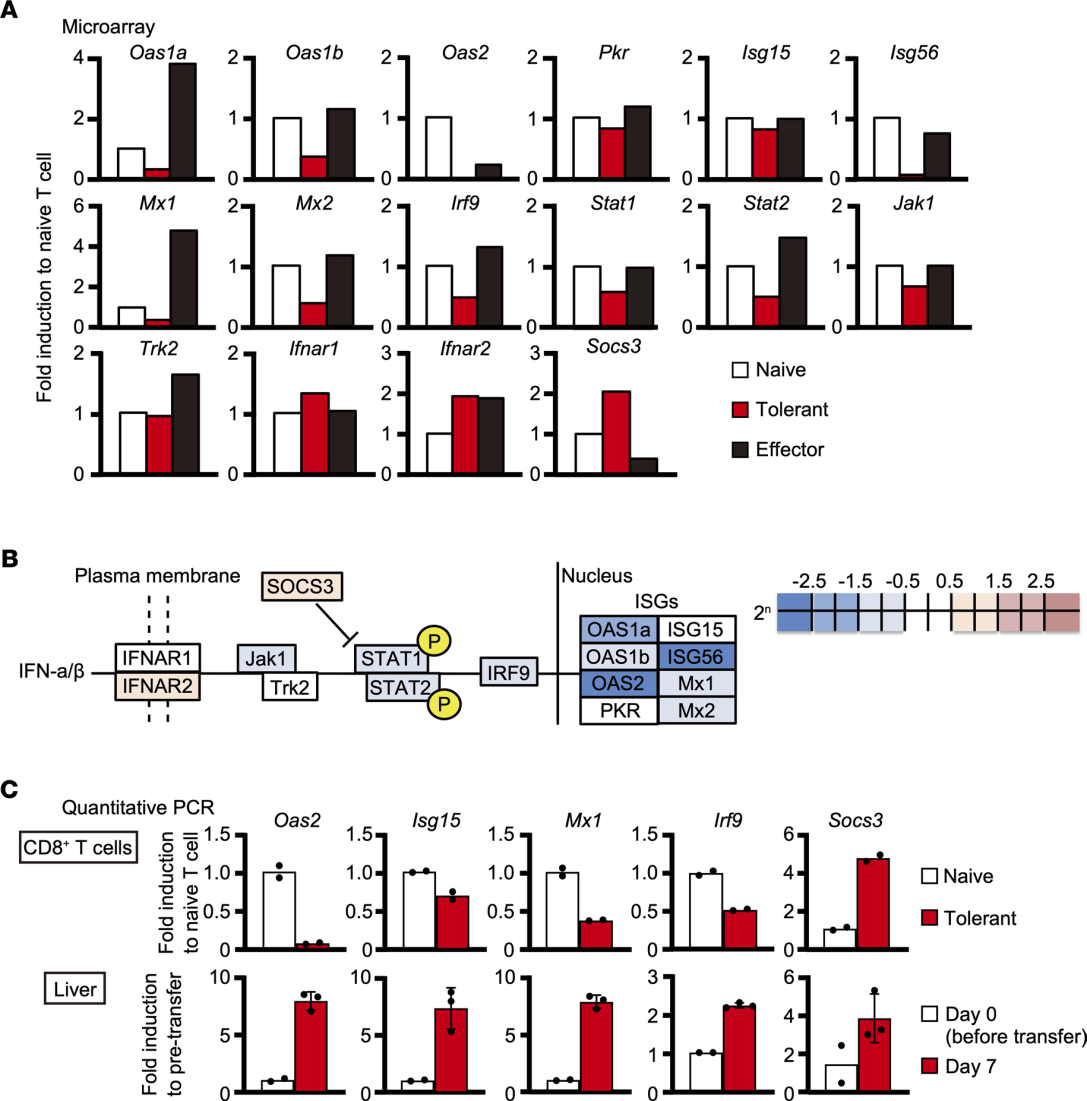
Type I IFN signaling is suppressed in tolerant CD8^+^ T cells. (**A** and **B**) The different expression levels of individual IFN-I–related genes among naive, tolerant, and effector T cells analyzed by microarray shown in (**A**) graphs and (**B**) a schema. (**C**) Selected gene expression levels were examined by reverse transcription quantitative PCR (RT-qPCR) in naive (*n* = 2) and tolerant (*n* = 2) COR93-specific T cells (upper panels) as well as the livers of HBV-Tg mice before (*n* = 2) and 7 days after (*n* = 3) T cell transfer (lower panels). Data represent mean ± SD (*n* ≥ 3).

We then analyzed the expression levels of individual genes related to IFN-I signaling. As shown in Figure 2A, the expression levels of many ISGs, such as 2′-5′ oligoadenylate synthetase 1A/1B (*Oas1a/1b*), *Isg56*, and MX dynamin-like GTPase 1/2 (*Mx1/2*), were decreased only in tolerant T cells, while those of some ISGs, such as *Isg15* and eukaryotic translation initiation factor 2-alpha kinase 2 (*Pkr*), were not. In addition, *Oas2* expression was severely decreased in tolerant T cells, although it was moderately decreased in effector T cells as well. Furthermore, the expression of genes associated with the IFN-I signaling pathway, such as IFN regulatory factor 9 (*Irf9*), *Jak1*, and *Stat1/2*, was also downregulated in tolerant T cells compared with naive T cells (Figure 2, A and B). Interestingly, the expression of *Socs3*, known to suppress IFN-I signaling, was upregulated in tolerant T cells. The changes in selected genes were validated by real-time quantitative PCR (Figure 2C). To determine whether these changes are restricted to HBV-specific T cells or reflect general responses in the liver, we compared the expression of these genes in whole livers from HBV-Tg mice before and on day 7 after adoptive transfer by quantitative PCR. As shown in Figure 2C, the expression levels of the selected IFN-I–related genes were increased in the whole liver after the T cell transfer. We also examined how long the expressions of IFN-I–related genes remained suppressed after intrahepatic priming by analyzing the expression of IFN-I–related genes in HBV-specific CD8^+^ T cells isolated from the livers of HBV-Tg mice on day 21 after adoptive transfer. As shown in Supplemental Figure 3, the expression of the genes that were downregulated on day 7 mostly remained suppressed on day 21. These results show that IFN-I signaling–related genes were continuously downregulated specifically in tolerant HBV-specific CD8^+^ T cells.

### IFN-I responsiveness is decreased in tolerant T cells.

**Figure 3:**
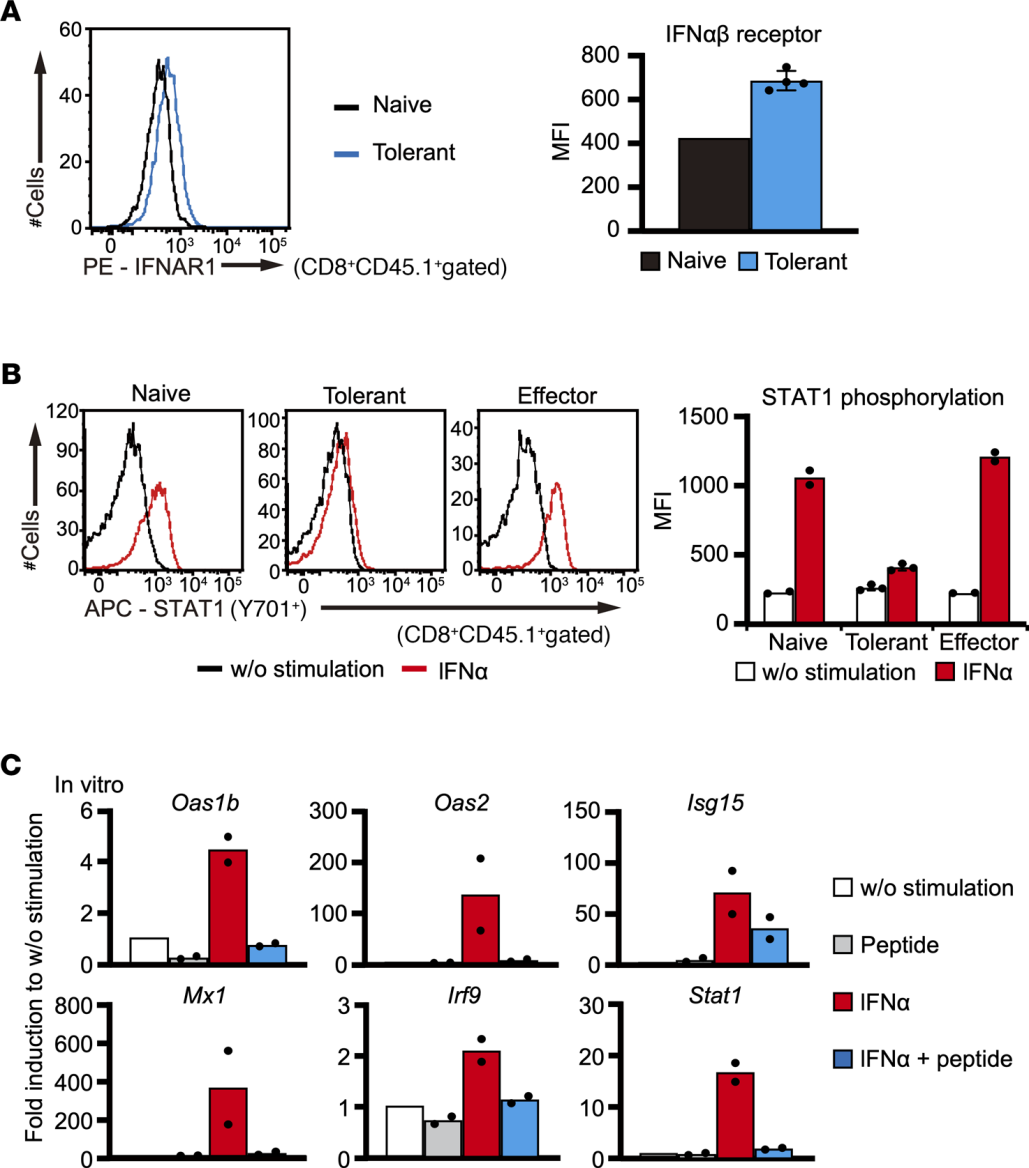
Responsiveness to IFN-α is suppressed in tolerant CD8^+^ T cells. (**A**) IFN-αβ receptor expression in naive (*n* = 1) and tolerant (*n* = 4) COR93-specific T cells was analyzed by FACS. (**B**) STAT1 phosphorylation was examined in naive (*n* = 2), tolerant (*n* = 3), and effector (*n* = 2) COR93-specific T cells after in vitro stimulation with IFN-α and COR93 peptide for 15 minutes. A representative result in each group is shown by histograms (left panels), while individual value and the average of mean fluorescence intensity in each group are shown by a dot plot and a bar graph, respectively (right panels). (**C**) The expression of IFN-I–related genes in COR93-specific CD8^+^ T cells were analyzed by RT-qPCR after stimulating spleen cells from COR93-specific TCR-Tg mice with IFN-α and/or COR93 peptide for 8 hours (*n* = 2 for each group). Data represent mean ± SD (*n* *≥* 3).

To elucidate molecular mechanisms regulating IFN-I signaling–related genes in dysfunctional CD8^+^ T cells, we compared the expression levels of IFN-I receptor and phosphorylated STAT1 between naive, effector, and tolerant T cells by flow cytometric analysis. The surface expression of the IFN-αβ receptor of tolerant T cells was not suppressed compared with that of naive T cells (Figure 3A), consistent with the mRNA expression (Figure 2A). Importantly, the expression of phosphorylated STAT1 after IFN-α stimulation for 15 minutes was lower in tolerant T cells compared with naive and effector T cells (Figure 3B), suggesting that IFN-I signaling was suppressed in tolerant T cells at a relatively proximal level. To examine the impact of TCR recognition on IFN-I responsiveness, we cultured naive COR93-specific CD8^+^ T cells with IFN-α in the presence or absence of COR93 peptide for 8 hours and analyzed the expression of IFN-I–related genes in CD8^+^ T cells. Surprisingly, ISGs as well as *Irf9* and *Stat1* expression were clearly suppressed by COR93 peptide addition in these T cells after IFN-α stimulation (Figure 3C). These results suggest that continuous TCR stimulation suppresses IFN-I signaling and its related gene expression.

### Robust activation of IFN-I signaling in the liver by poly(I:C) emulsified in lipid nanoparticles.

**Figure 4:**
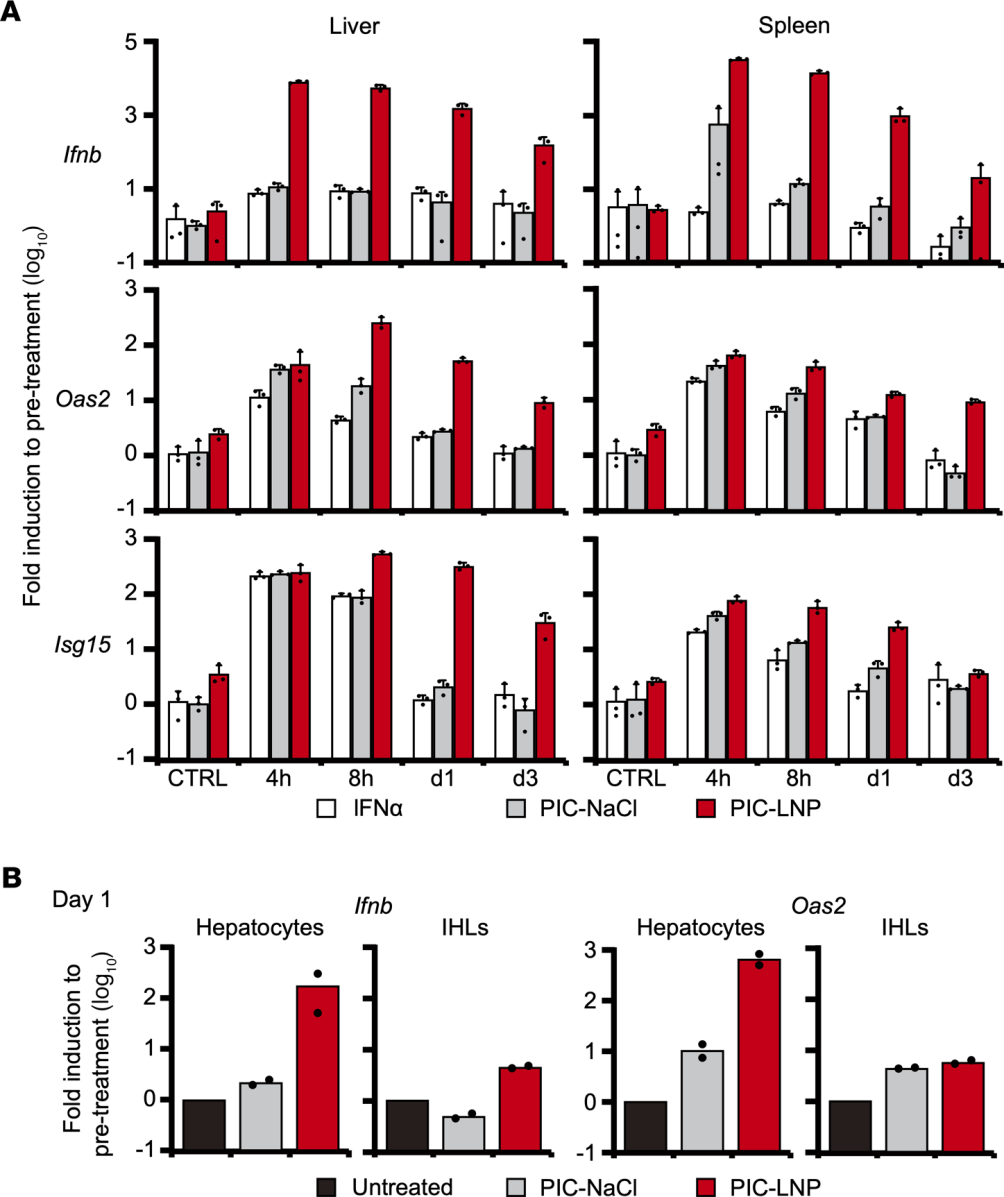
Strong induction of type I IFN responses in the liver by poly(I:C) emulsified in LNP. (**A**) B6 mice were treated with mouse IFN-α, poly(I:C) dissolved in NaCl (PIC-NaCl), or poly(I:C) emulsified in lipid nanoparticles (PIC-LNPs) and sacrificed at 4, 8, 24, or 72 hours after treatment (*n* = 3 for each group and time point). The mice were also sacrificed at 24 hours after injection of NaCl or LNPs to serve as controls. The mRNA expression levels of *Ifnb*, *Isg15*, and *Oas2* in the liver (left panels) and the spleen (right panels) were analyzed by RT-qPCR. (**B**) Hepatocytes and intrahepatic lymphocytes (IHLs) were isolated from the liver of B6 mice treated with PIC-NaCl and PIC-LNPs (*n* = 2 for each group) and analyzed for mRNA expression of *Ifnb* and *Oas2*. Data represent mean + SD (*n* *≥* 3).

The foregoing results indicate that intrahepatically primed tolerant T cells are relatively refractory to IFN-I signaling that is deemed required for functional differentiation of virus-specific CD8^+^ T cells. We, therefore, hypothesized that robust activation of IFN-I signaling in the liver is required to induce functional differentiation of HBV-specific CD8^+^ T cells. To activate intrahepatic IFN-I signaling, poly(I:C) was emulsified in commercialized lipid nanoparticles (PIC-LNPs) that were originally developed to deliver small interfering RNA to the liver, then intravenously administered to groups of B6 mice (30 μg/mouse). The mice were sacrificed at 4, 8, 24, and 72 hours after the treatment to determine the expression of *Ifnb*, *Oas2*, and *Isg15* mRNA in the liver and spleen. To determine the relative potency of PIC-LNP treatment to activate IFN-I responses in the liver, intrahepatic and splenic expression of these genes were also determined after administration of recombinant mouse IFN-α protein (100,000 IU/mouse) and poly(I:C) dissolved in NaCl (PIC-NaCl; 30 μg/mouse). As shown in Figure 4A, the induction of these genes was similar between the mice treated with IFN-α (white bars) and PIC-NaCl (gray bars). These treatments induced *Ifnb* mRNA only in the spleen, and *Oas2* and *Isg15* mRNA were transiently upregulated in the liver and spleen (Figure 4A). In contrast, PIC-LNPs induced 750 and 275 times more *Ifnb* mRNA expression in the liver and spleen, respectively, than PIC-NaCl at 4 hours after administration. Furthermore, high *Ifnb*, *Isg15*, and *Oas2* expression in the liver lasted more than 72 hours in the PIC-LNP–treated mice (Figure 4A, red bars). These results indicate the superior ability of PIC-LNPs to stimulate IFN-I signaling, especially in the liver.

To determine the cell population that induces IFN-β and ISGs’ expression in the liver after PIC-LNP treatment, we isolated hepatocytes and IHLs on day 1 after PIC-NaCl or PIC-LNP treatment and analyzed the expression of *Ifnb* and *Oas2*. Again, PIC-NaCl hardly induced the expression of these genes in hepatocytes or IHLs (Figure 4B, gray bars), while PIC-LNPs strongly did. Importantly, PIC-LNPs induced the expression of these genes more strongly in hepatocytes than in IHLs (Figure 4B, red bars), suggesting that the hepatocytes were the dominant target of IFN-I signaling activation by PIC-LNP treatment.

### Intrahepatic activation of IFN-I signaling induces functional HBV-specific T cells.

**Figure 5:**
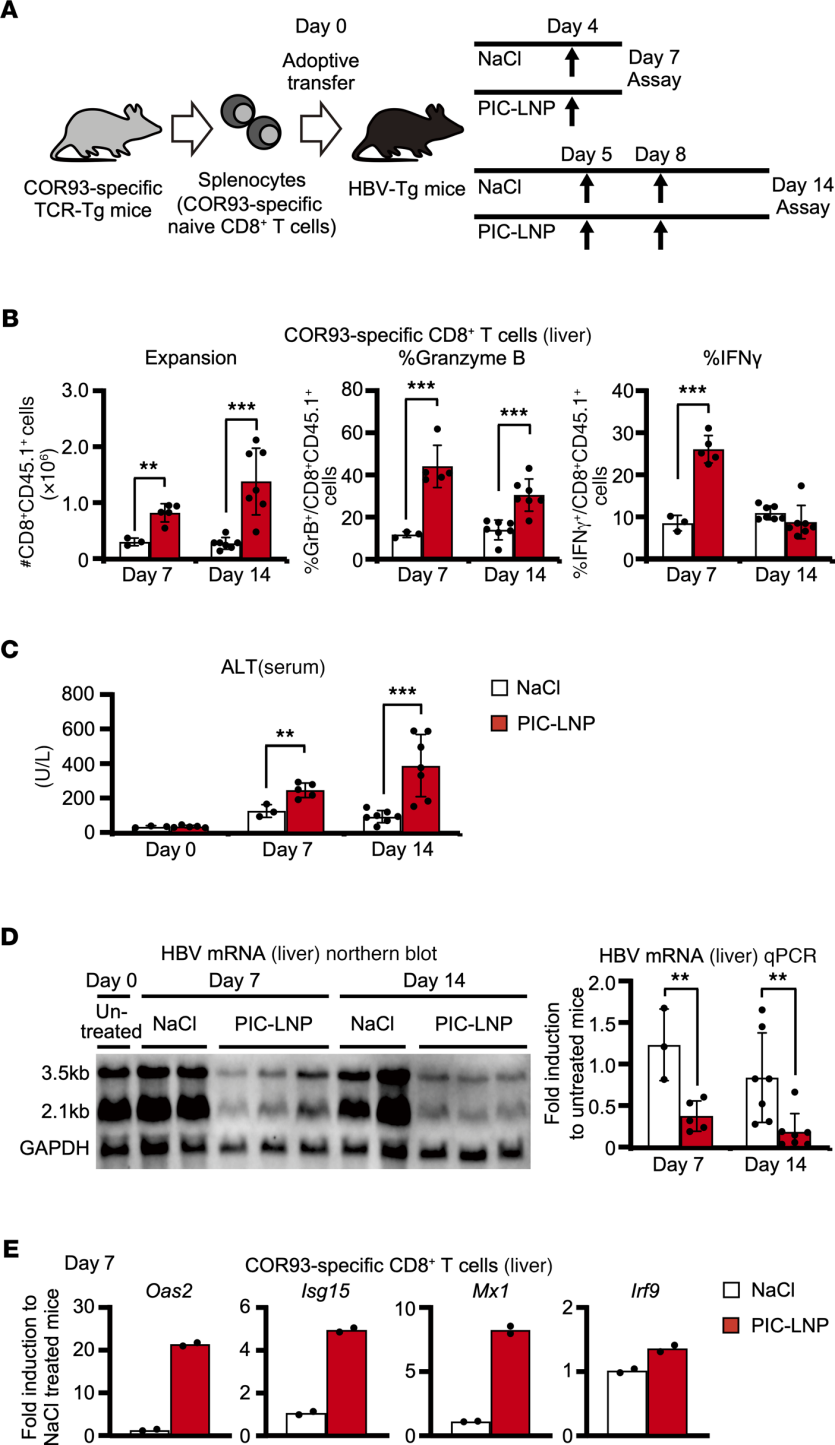
Intrahepatic activation of type I IFN response induces functional differentiation of HBV-specific CD8^+^ T cells and facilitates HBV clearance. (**A**) Schematic representation of the experiment (NaCl [day 7]; *n* = 3, PIC-LNP [day 7]; *n* = 5, NaCl [day 14]; *n* = 7, PIC-LNP [day 14]; *n* = 7). (**B**) The number of COR93-specific CD8^+^ T cells in the liver as well as their in vitro IFN-γ–producing ability and ex vivo granzyme B expression were analyzed by FACS. (**C**) Serum alanine aminotransferase (ALT) levels on days 0, 7, and 14 after transfer. (**D**) HBV mRNA expression in the liver analyzed by Northern blotting (left panel; representative samples) and RT-qPCR (right panel). (**E**) The mRNA expression levels of ISGs in COR93-specific CD8**^+^** T cells purified from the liver of HBV-Tg mice on day 4 after PIC-LNP or NaCl treatment (*n* = 2 for each group). Results are representative of 3 independent experiments. Data represent mean ± SD (*n* *≥* 3). *P* values determined by Student’s *t* test (1 sided, unpaired). *P < 0.05, **P < 0.01, ***P < 0.001.

Next, we examined whether IFN-I stimulation in the liver induces functional HBV-specific CD8^+^ T cell responses, as illustrated in Figure 5A. Four groups of HBV-Tg mice were adoptively transferred with naive COR93-specific CD8^+^ T cells. Two of them were treated with PIC-LNPs or NaCl on day 4 and sacrificed on day 7. The remaining groups were treated on days 5 and 8 and then sacrificed on day 14 after adoptive transfer. The number of intrahepatic HBV-specific T cells, as well as their cytokine-producing and cytotoxic ability, were analyzed. T cell responses were associated with serum alanine aminotransferase (ALT) activity and intrahepatic HBV mRNA expression. As shown in Figure 5B, HBV-specific CD8^+^ T cells vigorously expanded and differentiated into granzyme B–expressing cytotoxic effector cells in the PIC-LNP–treated mice on days 7 and 14. The frequency of IFN-γ–producing HBV-specific CD8^+^ T cells was higher in the PIC-LNP–treated group than the control group on day 7 (Figure 5B). Accordingly, serum ALT levels were moderately elevated in the PIC-LNP–treated mice (Figure 5C). Importantly, intrahepatic HBV mRNA expression on days 7 and 14 was decreased in the PIC-LNP group (Figure 5D). Furthermore, the expression of ISGs was restored in HBV-specific CD8^+^ T cells by PIC-LNP treatment (Figure 5E). These results suggest that strong IFN-I stimulation in the liver induces robust expansion and functional differentiation of HBV-specific CD8^+^ T cells in association with the restoration of IFN-I signaling in the T cells.

### Intrahepatic stimulation of IFN-I signaling enhances T cell responses irrespective of antigen specificity.

**Figure 6:**
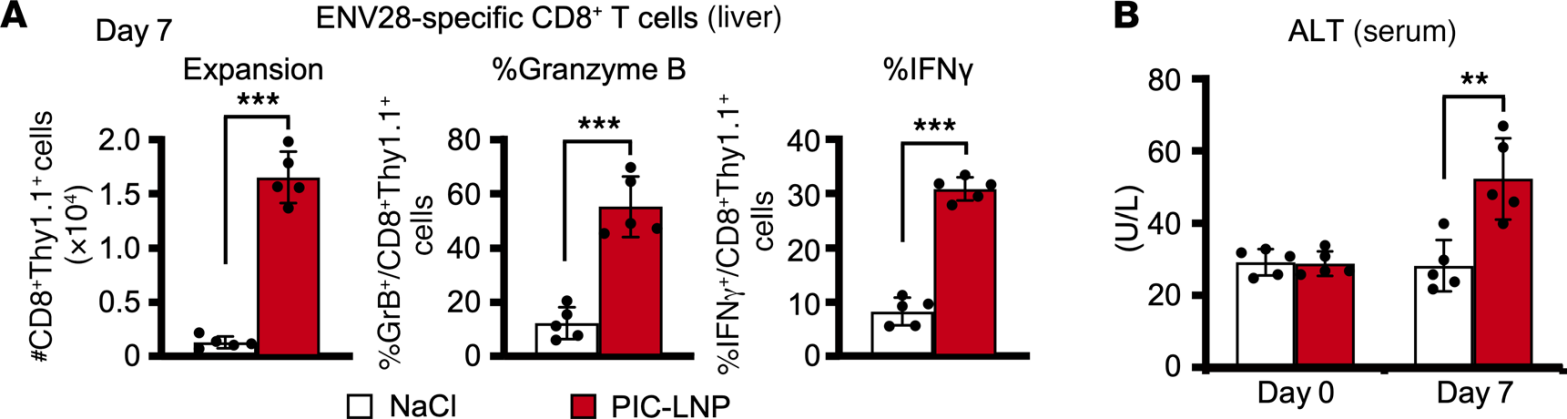
Activation of intrahepatic type I IFN signaling enhances HBV-specific CD8^+^ T cell responses irrespective of antigen specificity. HBV-Tg mice were adoptively transferred with ENV28-specific naive CD8**^+^** T cells and treated with PIC-LNPs or NaCl on day 4 (*n* = 5 for each group). (**A**) ENV28-specific CD8**^+^** T cell responses in the liver were analyzed on day 7 after transfer. (**B**) Serum ALT levels were measured on days 0 and 7. Results are representative of 2 independent experiments. Data represent mean ± SD. *P* values determined by Student’s *t* test (1 sided, unpaired). **P < 0.01, ***P < 0.001.

To examine whether enhanced HBV-specific T cell responses after PIC-LNP treatment are restricted to COR93-specific T cells, HBV-Tg mice were adoptively transferred with spleen cells harvested from another TCR-Tg mouse lineage that expresses TCR specific for an HBV surface antigen–derived (HBsAg-derived) epitope, ENV28. The recipient HBV-Tg mice were treated with PIC-LNP or NaCl on day 4 and sacrificed on day 7 after adoptive transfer to analyze intrahepatic T cell responses and serum ALT activity. As shown in Figure 6A, ENV28-specific CD8^+^ T cells expanded and differentiated to effector cells in PIC-LNP–treated mice. In association with the induction of T cell responses, serum ALT activity was increased in HBV-Tg mice that were treated with PIC-LNP (Figure 6B). However, the serum ALT elevation was rather modest, presumably due to the relatively small number of ENV28-specific CD8^+^ T cells in the liver compared with COR93-specific CD8^+^ T cells (Figure 5B and Figure 6A). Nevertheless, these results suggest that robust activation of IFN-I signaling in the liver enhances T cell responses irrespective of antigen specificity.

### IFN-I signaling in HBV-specific CD8^+^ T cells is required for the induction of T cell responses.

**Figure 7:**
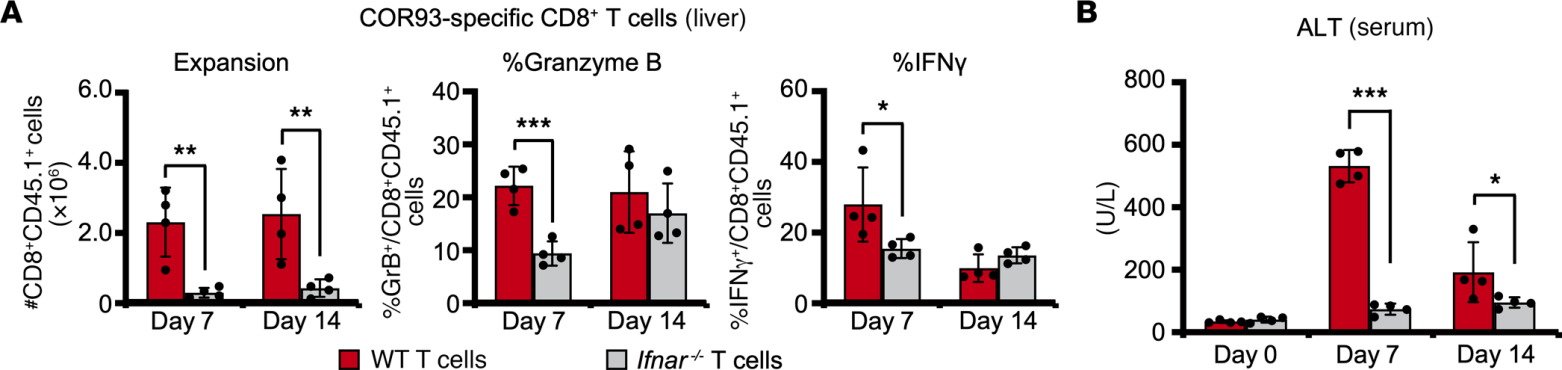
IFN-αβ receptor–deficient CD8^+^ T cells weakly expand and barely acquire the cytokine-producing and cytotoxic abilities. HBV-Tg mice were adoptively transferred with WT or *Ifnar^–/–^* COR93-specific CD8^+^ T cells on day 0 and treated with PIC-LNPs on day 4 and sacrificed on day 7, or treated on days 5 and 8 and sacrificed on day 14 (*n* = 4 for each group). (**A**) COR93-specific CD8^+^ T cell responses in the liver were analyzed on days 7 and 14. (**B**) Serum ALT levels were measured on days 0, 7, and 14. Results are representative of 2 independent experiments. Data represent mean ± SD. *P* values determined by Student’s *t* test (1 sided, unpaired). *P < 0.05, **P < 0.01, ***P < 0.001.

Finally, we examined whether the induction of HBV-specific CD8^+^ T cell responses after PIC-LNP treatment is dependent on IFN-I signaling in the T cells. COR93-specific TCR-Tg mice were crossed twice with IFN-I receptor–deficient (*Ifnar*^–/–^) mice, yielding *Ifnar*^–/–^ COR93-specific TCR-Tg mice. HBV-Tg mice were adoptively transferred with COR93-specific CD8^+^ T cells that were *Ifnar*^–/–^ or WT. The recipient mice were treated with PIC-LNPs on day 4 or days 5 and 8, and HBV-specific T cell responses and serum ALT levels were analyzed on day 7 or day 14, respectively, after adoptive transfer. As shown in Figure 7A, many fewer *Ifnar*^–/–^ COR93-specific T cells were detected in the liver than WT T cells on days 7 and 14 after adoptive transfer. The fractions of *Ifnar*^–/–^ T cells that acquired IFN-γ–producing and cytotoxic ability were also lower than WT T cells on day 7, although these differences between *Ifnar*^–/–^ and WT T cells were less obvious on day 14 (Figure 7A). In addition, serum ALT elevation was not detected after the adoptive transfer of *Ifnar*^–/–^ T cells (Figure 7B). These results suggest that PIC-LNP treatment enhances the expansion and functional differentiation of HBV-specific CD8^+^ T cells by directly acting on IFN-I signaling in HBV-specific CD8^+^ T cells.

## Discussion

The mechanistic basis of weak CD8^+^ T cell responses during persistent infection need to be elucidated to develop highly effective immunotherapy against chronic viral infections. Here, we performed transcriptome analyses on intrahepatically primed dysfunctional HBV-specific CD8^+^ T cells and found that the expression of ISGs was strongly suppressed. The level of phosphorylated STAT1 after IFN-α stimulation was reduced in intrahepatically primed CD8^+^ T cells compared with naive and effector T cells. In vitro results demonstrated a regulatory role of TCR stimulation in suppressing ISG induction. Finally, administration of LNP-coated poly(I:C) induced functional differentiation of intrahepatically primed HBV-specific CD8^+^ T cells in association with ISG upregulation in the T cells.

We initiated the current study to unravel early molecular events in HBV-specific CD8^+^ T cells that fail to acquire effector functions after intrahepatic priming. Intrahepatically primed HBV-specific CD8^+^ T cells exhibited a molecular signature that is very different from naive and effector CD8^+^ T cells. The expression of many checkpoint molecules was upregulated in intrahepatically primed dysfunctional T cells compared with naive and effector T cells (Supplemental Figure 2C), recapitulating the findings of persistent LCMV infection (3). However, there are many genes whose expression levels significantly differ between intrahepatically primed HBV-specific CD8^+^ T cells and exhausted LCMV-specific CD8^+^ T cells. Interestingly, the expression of various ISGs was strongly suppressed in the intrahepatically primed HBV-specific CD8^+^ T cells. During the preparation of this manuscript, the gene expression profile of intrahepatically primed HBV-specific CD8^+^ T cells was reported by another group (9). In that study, T cells from HBV-specific TCR-Tg mice were adoptively transferred into MUP-core transgenic mice in which only the HBV core antigen was expressed under the liver-specific promoter, and gene expression profile was analyzed using RNA sequencing. In contrast, we transferred the same TCR-Tg T cells into HBV-Tg mice that replicate HBV in the liver and produce infectious viral particles and examined gene expression profile by microarray analysis. Despite the differences, the gene expression profiles of intrahepatically primed CD8^+^ T cells are quite similar between the 2 studies. Upon close examination of the repository data by Bénéchet et al. (9), we confirmed that the expression of ISGs was also suppressed in the T cells that were primed in the liver of MUP-core transgenic mice. In stark contrast, ISG expression levels were strongly upregulated in LCMV-specific CD8^+^ T cells, according to the previous study by Wherry et al. (Supplemental Figure 4 and ref. 3), presumably due to the strong induction of IFN-Is during LCMV infection (10, 11). Contrary to LCMV, HBV is known to be a weak inducer of innate immune responses (12–14). Importantly, a comparison between this study and previously published studies that examined the gene expression profile of dysfunctional CD8^+^ T cells revealed that ISG expression was also downregulated in tumor-infiltrating CD8^+^ T cells (Supplemental Figure 4 and ref. 15). Thus, the suppression of IFN-I signaling may represent a general manifestation of T cells that are continuously exposed to an antigen in the absence of robust innate immune responses.

The physiological importance of reduced IFN-I signaling after intrahepatic priming is poorly understood. Ample evidence suggests that IFN-I signaling is essential for priming, expansion, and maintenance of antiviral CD8^+^ T cell responses. *Ifnar^–/–^* OT-I T cells proliferate in the presence of TCR stimulation and secondary signaling (i.e., CD80), but do not differentiate into effector T cells (16, 17). Abrogation of IFN-I signaling in virus-specific CD8^+^ T cells abolished their ability to expand and generate memory cells following LCMV infection (18). These observations formed a model that T cells require a third signal in addition to a primary (TCR) and secondary signal to differentiate fully, and IFN-Is are one group of the major cytokines that provide the third signal. Our data are consistent with this model. On the other hand, STAT1 downregulation in virus-specific CD8^+^ T cells was also observed during LCMV infection (19, 20), although its overall impact on ISG expression was not fully investigated. This STAT1 downregulation was postulated to curtail the negative impact of excessive IFN-Is on T cell responses (21). While suppression of IFN-I signaling may be beneficial in infections that induce strong IFN-I responses, such as LCMV (11, 22), the same mechanism can hinder CD8^+^ T cell responses when IFN-Is are not abundantly available. Reduction of IFN-I signaling in antigen-experienced T cells may represent another checkpoint machinery to maintain peripheral T cell tolerance. We do not know the exact mechanism of IFN-I signaling suppression in intrahepatically primed CD8^+^ T cells, either. At least in vitro, TCR signaling downregulates ISG expression by IFN-I stimulation. It remains to be elucidated whether reduced ISG expression in intrahepatically primed T cells reflects continuous TCR signaling. A number of studies indicate TCR signaling induces coinhibitory molecules such as PD-1, Tim-3, and Lag-3. A more recent study showed that TCR stimulation also upregulates a transcription factor’s expression, i.e., thymocyte selection-associated high mobility group box, which is shown to be a critical factor for T cell exhaustion (23). All these molecules were upregulated in the dysfunctional HBV-specific CD8^+^ T cells in this study (Supplemental Figure 2C). Further studies are necessary to determine whether any of these molecules contributes to the suppression of IFN-I signaling in intrahepatically primed T cells.

The current study has significant clinical implications as well. Although conventional and pegylated IFN-α have been first-line treatments for patients with chronic hepatitis B (CHB) (24, 25), the viral clearance rate of IFN-α treatments in CHB patients is rather low (26, 27). Clinical studies also showed that IFN-α treatment had little impact on HBV-specific CD8^+^ T cells (28). Reduced IFN-I signaling in intrahepatically primed T cells revealed in this study indicates that those T cells are relatively refractory to IFN-α treatments (Figure 2 and Figure 3). The local IFN-I concentrations sufficient to induce functionality in the T cells may be difficult to achieve by systemic administration without causing side effects. Indeed, a previous study demonstrated that systemic administration of pegylated IFN-α only transiently activates the JAK/STAT pathway in hepatocytes (29). Thus, our data argue for local and prolonged induction of IFN-Is for treating CHB. While our data suggest that strong activation of IFN-I signaling prevents exhaustion of newly generated T cells, the clinical significance of this study would be further strengthened if strong IFN-I stimulation could induce effector functions in exhausted CD8^+^ T cells that were exposed to the antigen for an extended period. In addition, because LNP-coated poly(I:C) strongly induces IFN-β not only in the liver but also in the spleen (Figure 4A), novel strategies that are more selective to the liver or intrahepatic T cells may be necessary to translate the findings in this study into clinics.

This study has a few limitations. First, the current study is limited to a mouse system and did not address whether HBV-specific CD8^+^ T cells also exhibit defective IFN-I signaling in patients with CHB. Second, the effect of intrahepatic stimulation of IFN-I was only tested at relatively early time points after T cell priming, while the majority of HBV-specific CD8^+^ T cells in patients with CHB have probably been exposed to HBV for a long period. Therefore, clinical value of this study is rather limited at this moment, and more experiments are required to test the therapeutic impact of intrahepatic IFN-I stimulation.

In summary, we have demonstrated that ISG expression is selectively suppressed in intrahepatically primed dysfunctional HBV-specific CD8^+^ T cells. The ISG suppression presumably reflects continuous TCR stimulation in the absence of IFN-Is and may serve as checkpoint machinery to maintain T cell tolerance. Our data suggest that induction of intrahepatic IFN-Is can rescue CD8^+^ T cells from functional exhaustion caused by hepatocellular priming and may have therapeutic value against chronic HBV infection.

## Methods

### Mice.

HBV-Tg mice (lineage 1.3.32, inbred B6, H-2b) have been previously described (30). Lineage 1.3.32 expresses all the HBV antigens and replicates HBV in the liver and kidney at high levels without any evidence of cytopathology. B6 mice (H-2b) were obtained from the breeding colonies of Japan SLC. In all experiments, the HBV-Tg and B6 mice were matched for age (8–10 weeks), sex (male), and serum hepatitis B e antigen levels in HBV-Tg mice before experimental manipulation. COR93 TCR-Tg mice (lineage BC10.3, inbred CD45.1, H-2b) and ENV28 TCR-Tg mice (lineage 6C2.16, inbred Thy1.1 BALB/c, H-2d) have been previously described (7, 31). Over 98% of the splenic CD8^+^ T cells of COR93 TCR-Tg mice recognize a Kb-restricted epitope located between residues 93 and 100 in the HBcAg (MGLKFRQL), while approximately 83% of the splenic CD8^+^ T cells of ENV28 TCR-Tg mice recognize an Ld-restricted epitope located between residues 28 and 39 of the HBsAg (IPQSLDSWWTSL). These T cells exhibit a naive T cell phenotype. For adoptive transfer experiments with ENV28-specific CD8^+^ T cells and HBV-Tg mice, 6C2.16 mice were mated once with B6 mice, and 1.3.32 mice were mated once with BALB/c mice. To generate *Ifnar*^–/–^ COR93-specific CD8^+^ T cells, BC10.3 mice were mated twice with *Ifnar*^–/–^ mice (H-2b, ref. 32), which were obtained from Oriental BioService.

### Adoptive transfer.

Spleen cells were isolated from BC10.3 mice or 6C2.16 × BALB/c F1 hybrids, and 2 × 10^7^ spleen cells per mouse were adoptively transferred into MHC class I–matched HBV-Tg or non-Tg recipients. In selected experiments, CD8^+^ cells were purified from the spleen cells of BC10.3 mice by negative selection using MACS (Miltenyi Biotec) according to the manufacturer’s instructions, and 3 × 10^6^ CD8^+^ T cells were adoptively transferred into each recipient. Effector HBV-specific CD8^+^ T cells were generated by adoptively transferring COR93-specific naive CD8^+^ T cells into B6 mice that were then immunized with COR93 peptide (purchased from Scrum) and a monoclonal anti-CD40 antibody (clone FGK4.5; catalog BE0016-2, Bio X Cell).

### Lymphomononuclear cell preparation.

IHLs and spleen cells were prepared as described previously (33). Livers were perfused with 10 mL of PBS via the portal vein to remove circulating lymphocytes, and the liver tissue was pressed through a 70 μm cell strainer (Corning) with the plunger of a 1 mL syringe and digested with 10 mL of RPMI 1640 medium (MilliporeSigma), containing 0.02% (*w/v*) collagenase IV (MilliporeSigma) and 0.002% (*w/v*) DNase I (MilliporeSigma), for 40 minutes at 37°C. Cell suspension was collected after removing hepatocytes and connecting tissues by slow-speed centrifugation (3 minutes, 20*g*). The cells were washed with RPMI 1640 and then overlaid on Percoll/Histopaque solution consisting of 12% Percoll (GE Healthcare, now Cytiva) and 88% Histopaque-1083 (MilliporeSigma). After centrifugation for 20 minutes at 750*g*, the IHLs were isolated at the interface. The lymphomononuclear cells were washed twice with RPMI 1640 medium and used for further analysis. Spleen cells were isolated by pressing through a 70 μm cell strainer, washed 3 times with RPMI 1640 medium containing 5% FBS, and used for further analysis. For adoptive transfer, spleen cells were further filtered out with a 70 μm cell strainer and washed with HBSS (MilliporeSigma) twice.

### Flow cytometric T cell analysis.

Lymphomononuclear cells isolated from the liver and spleen were incubated with an antibody mixture containing V450-conjugated anti-mouse CD45.1 (48-0453-82, eBioscience, Thermo Fisher Scientific), FITC-conjugated anti-mouse Thy1.1 (11-0900-85, eBioscience, Thermo Fisher Scientific), and V450-, FITC-, or PerCP-Cy5.5–conjugated anti-mouse CD8 (560469, 553031 [BD Biosciences], and 45-0081-80 [eBioscience, Thermo Fisher Scientific], respectively) for 1 hour on ice. IFN-αβ receptor was stained by incubating the cells with PE-conjugated anti-mouse IFNAR (12-5945-80, eBioscience, Thermo Fisher Scientific), followed by secondary staining with PE-conjugated anti-mouse IgG1 (550083, BD Biosciences). Intracellular cytokine staining was performed using Fixation/Permeabilization Solution Kit (BD Biosciences) after incubating for 5 hours at 37°C in the presence of brefeldin A and 2 μg/mL of COR93 or ENV28 peptide. Cells were then incubated with PE-conjugated anti-mouse IFN-γ (554412, BD Biosciences) and APC-conjugated anti-mouse granzyme B (GRB05, Invitrogen, Thermo Fisher Scientific) in 0.5% saponin. Staining for phosphorylated STAT1 (APC-conjugated anti-mouse STAT1pY701; 612597, BD Biosciences) was performed using Phosflow Perm Buffer III and Phosflow Lyse/Fix Buffer (BD Biosciences), following the manufacturer’s instructions. The cells were acquired using FACSCanto II (BD Biosciences), and the data were analyzed using FlowJo (FlowJo).

### Purification of COR93-specific CD8^+^ T cells and microarray analysis.

Lymphomononuclear cells isolated from the livers of HBV-Tg mice and spleens of immunized B6 mice were purified for CD8^+^CD45.1^+^ cells, i.e., COR93-specific CD8^+^ T cells, using MACS (Miltenyi Biotec). Total RNA was isolated from COR93-specific CD8^+^ T cells using RNeasy Mini Kit (QIAGEN) and submitted to DNA Chip Research Inc. for microarray analysis using the Agilent SurePrint G3 Mouse GE Microarray 8 x 60K Ver2.0 Chips (Agilent Technologies). Raw data were extracted with Agilent Feature Extraction Software (v11.5.1.1). Selected gProcessedSignal value was transformed by logarithm and normalized by the quantile method. Differentially expressed genes (DEGs) between each type of CD8^+^ T cells were identified with the limma package in R language (34). An adjusted *P* value was estimated using the Benjamini-Hochberg method. Significant DEGs were identified as those with |log_2_ fold change| > 1.5 and *P* < 0.01. Testing for functional enrichment of DEGs was performed using the Database for Annotation, Visualization, and Integrated Discovery online tool (35). Analyzed categories include GO terms (36) and Kyoto Encyclopedia of Genes and Genomes pathways (37). The GSEA was performed using the GSEA software (http://software.broadinstitute.org/gsea/) with 4762 gene set permutations using C2.all.v4.0.symbols (38). All data analysis and visualization of DEGs were conducted using R 3.5.1 (https://www.r-project.org). The microarray data discussed in this publication have been deposited in the National Center for Biotechnology Information’s Gene Expression Omnibus database (GSE159748).

### IFN-α and poly(I:C) treatment.

Mice were intravenously treated with 100,000 IU/mouse (approximately 5 MU/kg) of recombinant mouse IFN-α protein (Miltenyi Biotec) or 30 μg of poly(I:C) (MilliporeSigma). To deliver poly(I:C) to the liver, 30 μg/mouse of poly(I:C) was emulsified in the commercialized LNP (Invivofectamine 3.0; Invitrogen, Thermo Fisher Scientific) and intravenously injected following the manufacturer’s instructions. For in vitro experiments, IHLs and spleen cells were cultured with 2000 IU/mL of mouse IFN-α and 1 μg/mL of COR93 peptide before analysis.

### Isolation of primary hepatocytes.

Murine hepatocytes were isolated from B6 mice using a 2-step protocol as previously described (7) with some modification. The liver was perfused with Liver Perfusion Medium (Gibco, Thermo Fisher Scientific) for 4 minutes at a flow rate of 5 mL/min, then digested with 0.8 mg/mL type I collagenase (Worthington) in DMEM (Gibco, Thermo Fisher Scientific) for 8–12 minutes at 5 mL/min. Following complete digestion of the liver, cells were collected from the liver by disrupting the liver capsule and swirling the tissue in a Petri dish containing DMEM. The cell suspension was passed through a 100 μm cell strainer (Corning) and washed with DMEM repeatedly until the supernatant was no longer cloudy. Total RNA was extracted from isolated hepatocytes using RNeasy Mini Kit (QIAGEN) and used for RT-qPCR.

### RT-qPCR and Northern blotting.

Total RNA was isolated from liver tissue and T cells using ISOGEN (NIPPON GENE CO., LTD.) and RNeasy Mini Kit (QIAGEN), respectively, following the manufacturers’ instructions. Various mRNA levels were determined using RT-qPCR with StepOnePlus Real-Time PCR Systems (Applied Biosystems, Thermo Fisher Scientific). cDNA was synthesized using the High Capacity RNA-to-cDNA Kit (Applied Biosystems, Thermo Fisher Scientific). We used the predesigned TaqMan Gene Expression Assay (Applied Biosystems, Thermo Fisher Scientific) for the analyses of mRNA levels of mouse *Oas2*, *Isg15*, *Mx1*, *Irf9*, *Socs3*, and *Ifnb* as well as mouse *Gapdh* and *Cd8*, which were used for normalization. Intrahepatic HBV mRNA content was analyzed using primers HBV-166-F21 (5′-CACATCAGGATTCCTAGGACC-3′), HBV-344-R20 (5′-AGGTTGGTGAGTGATTGGAG-3′), and TaqMan probe HBV-242-F26FT (5′-FAM-CAGAGTCTAGACTCGTGGTGGACTTC-TAMRA-3′) (39). HBV mRNA content was also analyzed by Northern blotting, as described previously (31). Briefly, total RNA was separated in 1% agarose gel and transferred to a positively charged nylon membrane (Roche). The membrane was then hybridized with a DIG-dUTP–labeled, full-length HBV DNA fragment, which was generated using the DIG High Prime DNA Labeling and Detection Starter Kit II (Roche) and then detected by the alkaline phosphatase–labeled anti-DIG antibody according to the manufacturer’s instructions. The signals were analyzed using an Amersham Imager 680 (Cytiva).

### Biochemical analyses.

Liver injury was monitored by measuring serum ALT activity using Dri-Chem4000s (FUJIFILM Medical) following the procedure the manufacturer recommended.

### Statistics.

Student’s *t* test (1 sided, unpaired) was performed using Microsoft Excel. Data are depicted as the mean ± SD, and *P* values less than 0.05 were considered significant.

### Study approval.

All experiments involving mice were performed in the Center for Experimental Animal Science at Nagoya City University, following a protocol approved by the Institutional Animal Care and Use Committee of the Nagoya City University Graduate School of Medical Sciences (approved number H30M_33) and Animal Research: Reporting of In Vivo Experiments guidelines.

## Author contributions

MI conceived the study. KK, MI, and YT designed the study. KK, MI, and IB conducted experiments. KK, MI, SS, AN, TF, and YT analyzed the data. MO analyzed microarray data. MI, TF, and YT supervised experiments and provided resources. KK, MI, TF, and YT obtained financial support for this study. KK and MI wrote the manuscript. All authors revised the manuscript. KK and MI contributed equally to this work. KK is listed before MI because KK performed more experiments than MI, while MI mainly established the concept of this work.

## Supplementary Material

Supplemental data

## Version 1 01/05/2021

In-Press Preview

## Version 2 02/08/2021

Electronic publication
