# Supplementary material for: Restoration of type I interferon signaling in intrahepatically primed CD8+ T cells promotes functional differentiation
Source: JCI Insight. 2021 Feb 8;6(3):e145761. doi: 10.1172/jci.insight.145761 (PMC7934883; doi:10.1172/jci.insight.145761)
Supplement: Supplemental data [file jciinsight-6-145761-s123.pdf]

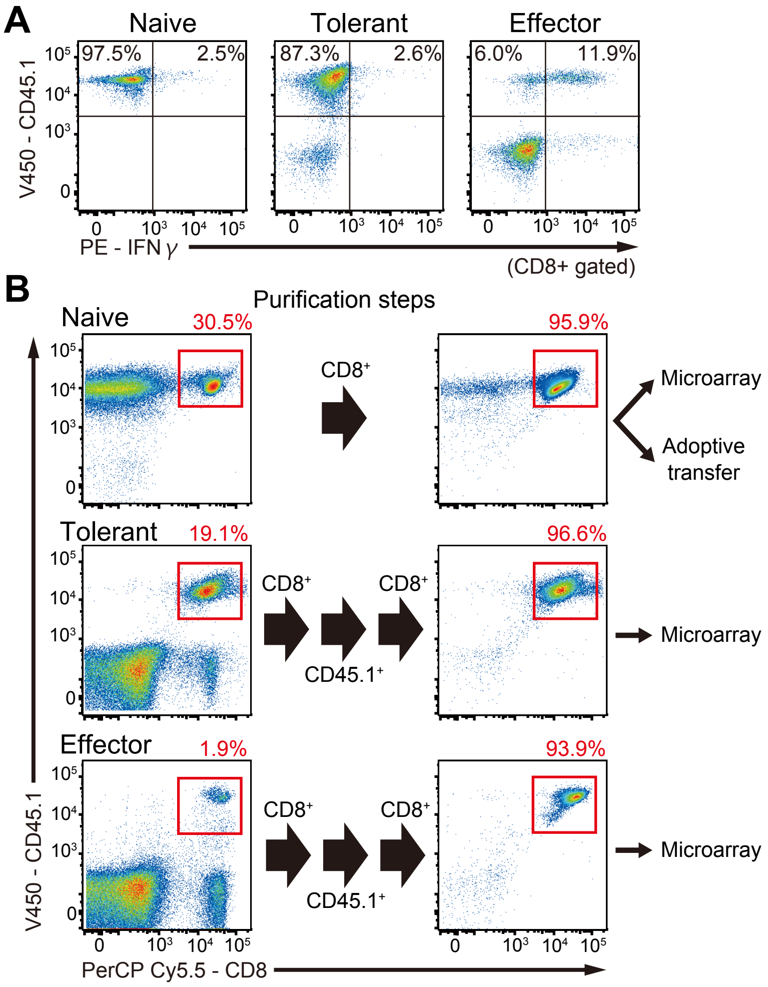

Supplemental Figure. 1 Purification of tolerant and effector COR93-specific CD8^{+} T cells. (**A**) Representative dot blots of in vitro IFNγ production in naive, tolerant, and effector COR93-specific CD8^{+} T cells. (**B**) The percentage of COR93-specific CD8^{+} T cells in total lymphocytes before (left) and after (right) purification procedures.

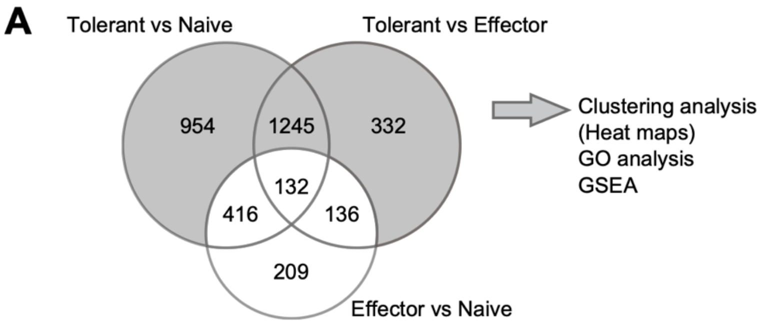

B Down-regulated gene sets in tolerant T cells

Tolerant vs Naive

NAME	NES	NOM p-val	FDR q-val
LI_INDUCED_T_TO_NATURAL_KILLER_DN	-1.9741	0.0000	0.0059
YU_MYC_TARGETS_DN	-1.8609	0.0000	0.0446
HADDAD_T_LYMPHOCYTE_AND_NK_PROGENITOR_DN	-1.7465	0.0000	0.2847
MELLMAN_TUT1_TARGETS_DN	-1.7138	0.0021	0.3657
LIAN_NEUTROPHIL_GRANULE_CONSTITUENTS	-1.7100	0.0111	0.3095
COATES_MACROPHAGE_M1_VS_M2_UP	-1.6923	0.0000	0.3362
STAMBOLSKY_TARGETS_OF_MUTATED_TP53_DN	-1.6820	0.0021	0.3284
DAVIES_MULTIPLE_MYELOMA_VS_MGUS_DN	-1.6596	0.0020	0.3815
HUANG_GATA2_TARGETS_UP	-1.6550	0.0000	0.3615
**HECKER_IFNB1_TARGETS**	**-1.6467**	**0.0064**	**0.3619**

Tolerant vs Effector

NAME	NES	NOM p-val	FDR q-val
**HECKER_IFNB1_TARGETS**	**-1.7374**	**0.0000**	**0.5138**
FARMER_BREAST_CANCER_CLUSTER_1	-1.7168	0.0018	0.3861
REACTOME_INHIBITION_OF_INSULIN_SECRETION_BY_ ADRENALINE_NORADRENALINE	-1.6684	0.0034	0.5729
REACTOME_ADP_SIGNALLING_THROUGH_P2RY1	-1.6531	0.0088	0.5568
REACTOME_SIGNAL_AMPLIFICATION	-1.6052	0.0070	0.8815
CAMPS_COLON_CANCER_COPY_NUMBER_DN	-1.5905	0.0091	0.8943
YU_MYC_TARGETS_DN	-1.5753	0.0177	0.9295
KEGG_LYSOSOME	-1.5695	0.0016	0.8722
BYSTRYKH_HEMATOPOIESIS_STEM_CELL_SCP2_QTL_TRANS	-1.5594	0.0160	0.8756
KEGG_PRIMARY_IMMUNODEFICIENCY	-1.5571	0.0144	0.8086

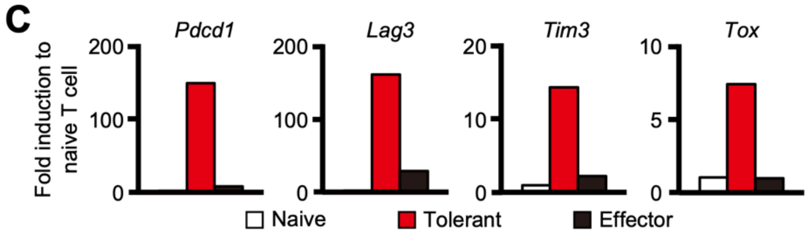

Supplemental Figure. 2 Supplemental data of microarray analysis. (**A**) Venn diagram represented the number of up- and down-regulated genes among naive, tolerant, and effector CPR93-specific CD8^{+} T cells. The genes in the gray area were uniquely up- and down-regulated in tolerant T cells and used for clustering, GO, and GSE analysis. (**B**) List of the top 10 enriched gene sets of down-regulated genes in tolerant COR93-specific CD8^{+} cells compared with naive and effector T cells, identified by GSEA (Molecular Signatures Database v6.2; C2 curated gene set). (**C**) The different mRNA expression levels of checkpoint molecules among naive, tolerant, and effector T cells.

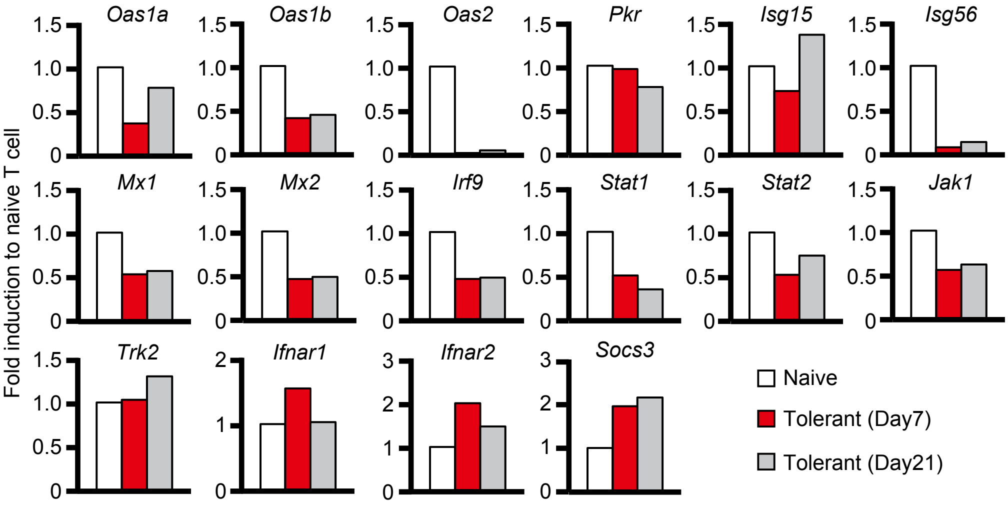

Supplemental Figure. 3 Expression of IFN-I signaling-related genes remains suppressed in tolerant T cells for a long period. The expression levels of IFN-I related genes were examined by microarray analysis in naive COR93-specific $CD8^+$ T cells as well as tolerant T cells on days 7 and 21 after transfer.

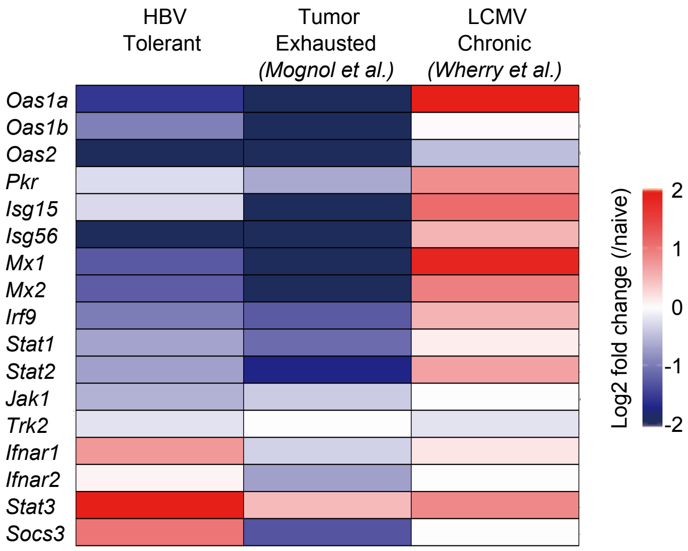

Supplemental Figure. 4 Comparison of IFN-I related genes with previous studies. Heat maps of IFN-I related genes were compared between the current study (HBV) and previous studies (OVA tumor; Mognol et al., 2017 and LCMV; Wherry et al., 2007).
